# Injecting drug use worsens the quality of life in HIV-HCV co-infected patients in Vietnam

**DOI:** 10.1038/s41598-026-46919-7

**Published:** 2026-04-09

**Authors:** Yoann Madec, Hanh Thi Hong Ngo, Tram Thi Phuong Pham, Nhung Thi Hong Le, Ha Thi Thu Nguyen, Jennifer Ilo van Nuil, Trang Trong Chu, Du Dinh Pham, Hieu Minh Tran, Hung Manh Lai, Linh Thi Thuy Doan, Tuan Anh Nguyen, Lan Trong Phan, Thang Hong Pham

**Affiliations:** 1 Epidemiology of emerging diseases unit, Institut Pasteur, Université de Paris, Paris, France; 2 HIV department, National Institute of Hygiene and Epidemiology, Hanoi, Vietnam; 3 Oxford University Clinical Research Unit, Ho Chi Minh City, Vietnam; 4 Nuffield Department of Medicine, University of Oxford, Oxford, UK; 5 Center for diseases control Yen Bai province, Yen Bai City, Vietnam; 6 Center for diseases control Nghe An province, Vinh, Vietnam; 7 Vietnam Administration for Disease Prevention, Hanoi, Vietnam

**Keywords:** HIV, Hepatitis C, People who inject drugs, heath-related quality of life, Depression, Diseases, Health care, Medical research, Psychology, Psychology

## Abstract

In Vietnam, in 2024, the HIV prevalence was estimated at 0.4% accounting for approximatively 270,000 adult people living with HIV (PLHIV). PLHIV may also face hepatitis C virus (HCV) co-infection, although co-infection disproportionally affects people who inject drugs (PWID). It has been shown that usually, PLHIV have lower health-related quality of life (HRQoL) than HIV-negative individuals. This study, focusing on HIV-HCV co-infected individuals on antiretroviral therapy (ART), offers the opportunity to investigate their HRQoL and compare PWID to non-PWID. The MOVIDA Hep 2 project, a prospective cohort study, enrolled HIV-HCV co-infected patients on ART in Vietnam. At enrolment, the HRQoL in the cohort was assessed using the EQ-5D-5L standardized scale. Low self-rated HRQoL was defined as providing a score below the 25^th^ percentile. Factors associated with this outcome were identified using mixed-effects logistic regression models. A total of 343 HIV-HCV co-infected participants were enrolled in the study, of whom 249 (72.6%) were PWID. Participants were on ART for 116 months in median. Overall, 62 (18.1%) participants reported pain/discomfort and 42 (12.2%) reported anxiety/depression, the proportions did not differ by PWID status (p=0.85 and p=0.07, respectively; Fisher exact test). However, when self-rating their HRQoL, PWID gave a significantly lower score (p<0.001). Factors associated with low self-rated HRQoL were PWID status, ethnicity and alcohol consumption as well as reporting pain/discomfort and anxiety/depression.  Interestingly, PWID self-rated their HRQoL at a lower level than non-PWID despite similar long the duration of ART (around 10 years in median) in both groups. Our results suggest that PWID face more physical and psychological distress. The extent of such problems is difficult to ascertain, but screening patients to identify those who need more specific support or attention would be beneficial. Offering integrated mental health screening and care could also benefit these patients, but then the question of integrating mental health care in the national health insurance must also be questioned or addressed.

**Supplementary Information:**

The online version contains supplementary material available at 10.1038/s41598-026-46919-7.

## Introduction

In Vietnam, HIV prevalence was estimated at 0.4% in the adult population in 2024 accounting for approximatively 270,000 adult people living with HIV (PLHIV)^1^. Antiretroviral therapy (ART) has been available from 2005, and the test-and-treat strategy following the 2016 WHO guidelines^2^ was implemented since 2017^3^.

In the last years, HIV prevalence declined in people who inject drugs (PWID) in Vietnam, from 14.0% in 2018^4^ to 9.1 in 2024^1^. However, PWID still represent a significant proportion among PLHIV. In a recent cohort study implemented in 6 provinces in North Vietnam, PWID represented about 50% of PLHIV initiating ART^5^.

It has been shown that usually, PLHIV have lower health-related quality of life (HRQoL) than HIV-negative individuals^6^. A study in Vietnam showed, however, that QoL significantly improved on ART^7^. For the well-being of PLHIV but also to improve health outcomes, holistic care should be considered. HRQoL and mental well-being are therefore essential as they could impact use of HIV care services and treatment adherence. In that respect, more attention should be given to HRQoL in PLHIV.

In previous evaluations in PLHIV in Vietnam, 14% to 20% suffered from depression^8,9^, but only 2% suffered from severe depression^9^. In these evaluations, the association between depression and PWID status or ART duration were however not investigated. Yet, it is suggested that, in Vietnam, drug-related stigma is more important than HIV-related stigma^10^. Stigma being in turn associated with poorer health outcomes^11^. On the contrary, a study in China found that family support was associated with improved QoL^12^. This raises the question of whether this translates to deteriorated HRQoL in PWID as compared to other PLHIV. Poorer quality of life could induce lower adherence to medication and translate into poorer health outcomes. In a previous evaluation in Vietnam, we showed that PWID were at higher odds of virological failure^13^ and higher odds of death^14^.

The present study aims at describing HRQoL in PLHIV co-infected with HCV on ART for several years, and comparing the HRQoL between PWID and non-PWID.

## Methods

The MOVIDA Hep 2 prospective cohort study enrolled HIV-HCV co-infected patients in two provinces in North Vietnam, Nghe An and Yen Bai, from December 2023 to November 2024. Of the 10 participating care sites, all were district level. Criteria for enrolment were: HIV-HCV co-infection, age ≥ 18 years, never having received treatment for HCV, and providing informed consent. HCV testing was performed in patients who were never tested before and in those who had received a negative HCV result more than 1 year prior to enrolment. The MOVIDA Hep 2 study was approved by Institutional Review Board at Institut Pasteur (Paris, France) and at the NIHE (Hanoi, Vietnam). All participants provided written informed consent. All experiments were performed following published guidelines, regulations and the declaration of Helsinki.

The main goal of the MOVIDA Hep 2 study was to compare HCV treatment outcomes between PWID and non PWID. In Vietnam, PWID status is documented in the national medical record file, although it does not distinguish current or former drug use. Health-care staff in HIV clinics are therefore familiar with injecting drug use and trained to document it reliably. Furthermore, at enrolment in the study, all participants were questioned by health-care staff about their current or former drug use, and enrolment in a drug-substitution programme.

At enrolment, HRQoL was assessed using the EQ-5D-5 L tool^15,16^, which consists of five questions evaluating five health dimensions (mobility, self-care, usual activities, pain/discomfort, anxiety/depression) and a visual analog scale (VAS). The ED-5D-5L instrument was completed by the clinical staff who asked the questions to the study participants. For each of the five questions, five possible answers were offered to the participant from no issue to stronger issue. For the VAS, participants were asked to self-rate their general health between 0 (worse) and 100 (best). This scale is validated in Vietnamese language and has been used in various populations before^17–20^.

We evaluated liver fibrosis (as a proxy for liver damage) using the non-invasive APRI (aspartate aminotransferase (AST) to platelet ratio index) and FIB-4 (Fibrosis-4) scores^21–23^. APRI is calculated from AST and platelet counts, whereas FIB-4 incorporates age of the subject, AST, alanine aminotransferase (ALT), and platelet counts.

### Statistical analysis

First, patients’ characteristics at enrolment were compared between PWID and non-PWID. The chi-2 or Fisher exact test were used for categorical variables, depending on sample size. The student t-test was used for continuous variables. In subsequent analyses, based on information obtained from the participants themselves, we further distinguished PWID as: former PWID, PWID enrolled in drug-substitution programme, and current PWID.

The proportion of participants presenting issues (at any severity level) in each of the five dimensions addressed by the EQ-5D-5L scale, as opposed to those presenting no issue, was estimated and compared between PWID and non-PWID using a Fisher exact test. The overall EQ-5D-5L score were estimated using the value set validated for Vietnam that attributes a score to each profile^24^. This EQ-5D-5L score was compared between PWID and non-PWID using the Kruskal-Wallis non-parametric rank test as the score did not follow a gaussian distribution. The self-rated health level was also compared between PWID and non-PWID using the Kruskal-Wallis non-parametric rank test as the VAS-score did not follow a gaussian distribution. Low self-rated HRQoL was defined as a VAS-score below the 25th percentile. As no validated threshold have been reported in the literature, this threshold was chosen empirically as it allows to define two groups with sample size allowing analyses.

Factors associated with (i) pain/discomfort (any level versus none), (ii) anxiety/depression (any level versus none) and (iii) low self-rated HRQoL (score < 25th percentile versus ≥ 25th percentile) were identified using mixed effects logistic regression models, the random-effect component of the models consisted in a random intercept to account for potential correlation between subjects from a same clinical site. In these analyses, effect of PWID status was investigated using a binary (PWID and non-PWID) and a four-category (former PWID, PWID enrolled in a drug-substitution programme, current PWID and non-PWID) classification. The other factors investigated were demographic characteristics (gender, age, ethnicity, marital status, education level), time since ART initiation and health-related factors (body mass index (BMI), hepatitis B (HBV) status, HCV viral load level, APRI and FIB4 score) and alcohol consumption. Reporting pain/discomfort signs, reporting anxiety/depression signs and low self-rated HRQoL were also considered in these analyses. Factors associated with the outcome in univariate analysis with a p-value < 0.20 were considered in the multivariate analysis, however, gender and age were forced in the multivariate model regardless of their p-value in univariate analysis. Then, factors independently associated with the outcome were identified using a stepwise backward procedure. Of note, when a category accounted for 0 event, it was merged with an adjacent category (see below Tables 4 and 5). Without this recategorization, the subjects for the category with no outcome would have been excluded from analysis which could have led to selection bias. The category was merged with the previous one and then with the following one, to assess whether results differed. This applied only if the category did not correspond to missing values.

All analyses were performed using Stata version 17 (Stata Corp., Texas, USA).

## Results

Of 714 patients screened, 296 tested HCV-negative, 75 tested HCV-positive but declined participation, and 343 HIV-HCV co-infected patients were enrolled in the study. At study enrolment, all participants were naïve of HCV treatment. Overall, 249 (72.6%) participants were PWID (Table 1). When further questioned about drug use, 198 (79.5%) declared former drug use, 32 (12.9%) declared being enrolled in a substitution programme, and 19 (7.6%) declared being current PWID.

Overall, most participants were male, but all of the PWID were male (Table 1). Median (inter quartile range (IQR)) age at enrolment was 41 (36–47) years. At enrolment, all patients were on ART. The median (IQR) time since ART initiation was 109 (87–138) months while the median (IQR) time since HIV diagnosis was 116 (90–146) months. Most participants had never been tested for HBV; and when tested only 2.9% were found to be infected with HBV on top of HIV and HCV.

**Table 1:** Participants’ characteristics at enrolment.

	All (N=343)	PWID (N=249)	Non-PWID (N=94)	P
Male gender, n (%)	324 (94.5)	249 (100)	75 (79.8)	<0.001
Age (years)				0.87
Median (IQR)	41 (36–47)	42 (36–47)	41 (37–45)	
BMI (kg/m²)				0.48
Median (IQR)	20.4 (19.3–22.0.3.0)	20.3 (19.2–22.0.2.0)	20.8 (19.4–22.0.4.0)	
BMI categories (kg/m²), n (%)				0.83
<17	10 (2.9)	8 (2.8)	2 (3.6)	
17–18.5.5	43 (12.5)	35 (12.2)	8 (14.5)	
18.5–25.5	277 (80.8)	234 (81.2)	43 (78.3)	
25–30	9 (2.6)	8 (2.8)	1 (1.8)	
≥30	4 (1.2)	3 (1.0)	1 (1.8)	
HBV diagnosis, n (%)				0.06
Negative	132 (38.5)	118 (41.0)	14 (25.5)	
Positive	10 (2.9)	8 (2.8)	2 (3.6)	
Not tested	201 (58.6)	162 (56.2)	39 (70.9)	
Time since HCV diagnosis (months)				0.55
Median (IQR)	0 (0–3.8.8)	0 (0–3.2.2)	0 (0–5.5.5)	
HCV viral load				0.65
Undetected	57 (16.6)	40 (16.1)	17 (18.1)	
Detected	286 (83.4)	209 (83.9)	77 (81.9)	
Ethnicity, n (%)				0.019
Thai	204 (59.5)	145 (58.2)	59 (62.8)	
Kinh	94 (27.4)	77 (30.9)	17 (19.1)	
Other	45 (13.1)	27 (10.8)	18 (19.1)	
Marital status, n (%)				0.039
Single	67 (19.5)	55 (22.1)	12 (12.8)	
Married/partnered	240 (70.0)	172 (69.1)	68 (72.3)	
Divorced/Separated	30 (8.7)	20 (8.0)	10 (10.6)	
Unknown	6 (1.8)	2 (0.8)	4 (4.3)	
Education level, n (%)				0.16
No schooling	23 (6.7)	17 (6.8)	6 (6.4)	
Elementary	92 (26.8)	59 (23.7)	33 (35.1)	
Secondary	148 (43.2)	110 (44.2)	38 (40.4)	
High school	72 (21.0)	58 (23.3)	14 (14.9)	
College/university	8 (2.3)	5 (2.0)	3 (3.2)	
Time since HIV diagnosis (months)				0.15
Median (IQR)	116 (90–146)	120 (92–147)	106 (78–139)	
Time since ART initiation (months)				0.36
Median (IQR)	109 (87–138)	111 (90–140)	104 (77–136)	
ART combination, n (%)				0.69
3TC-TDF-DTG	322 (94.1)	233 (93.9)	89 (94.6)	
Other DTG-based	3 (0.9)	3 (1.2)	-	
3TC-TDF-EFV/NVP	15 (4.4)	11 (4.4)	4 (4.3)	
PI-based	2 (0.6)	1 (0.4)	1 (1.1)	
APRI, n (%)				0.51
≤0.5	113 (32.9)	82 (32.9)	31 (33.0)	
0.5 to 1.5	118 (34.4)	87 (35.0)	31 (33.0)	
1.5 to 2.0	15 (4.4)	13 (5.2)	2 (2.1)	
≥2.0	28 (8.2)	21 (8.4)	7 (7.4)	
Missing	69 (20.1)	46 (18.5)	23 (24.5)	
FIB-4 score, n (%)				0.18
<1.3	120 (35.0)	91 (36.6)	29 (30.8)	
1.3 to 2.67	102 (29.7)	79 (31.7)	23 (24.5)	
2.67 to 3.25	15 (4.4)	8 (3.2)	7 (7.4)	
>3.25	37 (10.8)	25 (10.0)	12 (12.8)	
Missing	69 (20.1)	46 (18.5)	23 (24.5)	
Alcohol consumption				0.013
No	112 (32.6)	71 (28.5)	41 (43.6)	
Yes	230 (67.1)	177 (71.1)	53 (56.4)	
Missing	1 (0.3)	1 (0.4)	-	

*PWID* people who inject drugs; IQR inter quartile range; *HBV* hepatitis B virus; *ART* antiretroviral therapy; *HCV* hepatitis C virus; *BMI* body mass index; *APRI* aspartate aminotransferase to platelet ratio index.

The number of PLHIV reporting mobility or physical issues, captured by the EQ-5D-5L scale, was limited (Table 2). In most participants, these issues were slight to moderate.

Overall, 62 (18.1%) and 42 (12.2%) participants reported pain/discomfort and anxiety/depression signs at any severity level, respectively.

Overall, five participants reported severe signs (the two highest severity level) in at least one dimension covered by the EQ-5D-5L scale (data not shown in Table). All these patients were PWID (4 former PWID and 1 enrolled in a drug substitution programme); however, the comparison with non-PWID (none reported severe signs) was not significant (*p* = 0.36). Two participants reported severe anxiety/depression signs, one also reported light mobility issue while the other one reported no issue in the four other dimensions. One participant reported severe pain/discomfort as well as severe signs in mobility, self-care and usual activities, but moderate anxiety/depression. The last two reported severe mobility, self-care and usual activities issues, and slight or moderate pain/discomfort and anxiety/depression.

Six participants (three PWID and three non-PWID) did not complete the VAS to self-rate their general health at enrolment. Overall, VAS scores differed significantly across the four PWID status groups (Kruskall-Wallis test: *p* = 0.002; Table 2). Post-hoc pairwise comparisons were conducted using a Dunn’s test with Bonferroni correction. Current PWID had significantly lower scores as compared to non-PWID, to former PWID and to PWID enrolled in a drug substitution programme (*p* < 0.001, *p* < 0.001 and *p* = 0.038, respectively). PWID enrolled in a drug-substitution programme had significantly lower score than non PWID (*p* = 0.025). All other comparisons were not significant.

**Table 2:** Assessment of the health-related quality of life using the EQ-5D-5 L scale.

	All (*N* = 343)	PWID, former (*N* = 198)	PWID, substitution (*N* = 32)	PWID, current (*N* = 19)	Non-PWID (*N* = 94)	*p*
Mobility issue, *n* (%)	25 (7.3)	17 (8.6)	1 (3.1)	1 (5.3)	6 (6.4)	0.82
Self-care issue, n (%)	8 (2.3)	6 (3.1)	1 (3.1)	1 (5.3)	–	0.17
Usual activities issue, n (%)	10 (2.9)	5 (2.5)	1 (3.1)	1 (5.3)	3 (3.2)	0.63
Pain/discomfort, n (%)	62 (18.1)	36 (18.2)	4 (12.5)	3 (15.8)	19 (20.2)	0.79
Anxiety/depression, n (%)	42 (12.2)	20 (10.1)	5 (15.6)	6 (31.6)	11 (11.7)	0.07
Global score*, median (IQR)	1 (0.94–1.94)	1 (1–1)	1 (1–1)	1 (1–1)	1 (0.93–1.93)	0.74**
VAS score						0.002**
N (%)	337 (98.2)	196 (99.0)	31 (96.9)	19 (100)	91 (96.8)	
Median (IQR)	86 (80–91)	86 (80–92)	82 (78–88)	70 (70–80)	90 (80–93)	

*PWID* people who inject drugs; *IQR* inter quartile range; *VAS* visual analog scale. *Vietnam standardized **Kruskal-Wallis test.

Factors associated with pain/discomfort were identified using univariate and multivariate mixed effect logistic regression models. The factors that were significantly associated with pain/discomfort, both in univariate and multivariate analysis, were age, reporting anxiety/depression signs and a VAS score < 25th percentile (Table 3). As compared to participants aged 30–39 years, those aged ≥ 50 years were at significantly higher odds of reporting pain/discomfort (adjusted odds ratio (aOR) [95% confidence interval (CI)]: 7.50 [2.61–21.51]). As compared to those who did not report anxiety/depression signs, those who reported anxiety/depression signs were at higher odds of reporting pain/discomfort (aOR [95% CI]: 5.95 [2.36–14.98]). Those with a VAS score < 25th percentile, as compared to those with a higher score, were at higher odds of reporting pain/discomfort (aOR [95% CI]: 7.14 [3.40–15.01.40.01]). BMI and ethnicity were no longer associated with pan/discomfort after adjusting for age, reporting anxiety/depression signs and a VAS score < 25th percentile. PWID status was not associated with self-report of pain/discomfort, nor was the level of liver damage as expressed by APRI and FIB4 scores, or alcohol consumption.

**Table 3:** Factors associated with pain/discomfort (mixed effect logistic regression).

	N	Pain/discomfort	Crude OR (95% CI)	P	Adj. OR (95% CI)	P
PWID				0.65		
No	94	19 (20.2)	1			
Former	198	36 (18.2)	0.91 (0.48-1.74)			
Drug-substitution prog.	32	4 (12.5)	0.50 (0.15-1.64)			
Current	19	3 (15.8)	0.61 (0.15-2.47)			
Male gender				0.98		
No	19	4 (21.5)	0.98 (0.30-3.17)			
Yes	324	58 (17.9)	1			
Age (years)				<0.001		0.001
≤29	14	2 (14.3)	1.41 (0.27-7.20)		3.24 (0.56-18.90)	
30-39	126	12 (9.5)	1		1	
40-49	150	26 (17.3)	2.33 (1.06-5.12)		1.92 (0.76-4.84)	
≥50	53	22 (41.5)	7.60 (3.08-18.73)		7.50 (2.61-21.51)	
BMI (kg/m²)				0.004		
<18.5	53	19 (35.9)	3.09 (1.57-6.09)			
18.5-25	277	40 (14.4)	1			
≥25	13	3 (23.1)	2.06 (0.50-8.39)			
HBV status				0.64		
Negative	132	25 (18.9)	1			
Positive	10	1 (10.0)	0.41 (0.05-3.57)			
Not tested	201	36 (17.9)	0.81 (0.40-1.62)			
HCV viral load				0.26		
Undetected	57	13 (22.8)	1			
Detected	286	49 (17.1)	0.66 (0.32-1.36)			
Ethnicity				0.02		
Thai	204	26 (12.7)	1			
Kinh	94	28 (29.8)	2.94 (1.37-6.30)			
Other	45	8 (17.8)	1.61 (0.61-4.26)			
Marital status				0.46		
Single	67	8 (11.9)	1			
Married/partnered	240	47 (19.6)	1.88 (0.82-4.31)			
Divorced/Separated	30	5 (16.7)	1.66 (0.46-5.99)			
Unknown	6	2 (33.3)	2.89 (0.42-19.86)			
Education level				0.16		
No schooling	23	7 (30.4)	3.26 (1.13-9.45)			
Elementary	92	16 (17.4)	1.39 (0.66-2.91)			
Secondary	148	21 (14.2)	1			
High school or higher	80	18 (22.5)	1.53 (0.73-3.20)			
Time on ART				0.52		
≤60 months	56	9 (16.1)	1			
60-120 months	147	23 (15.6)	1.00 (0.42-2.37)			
>120 months	140	30 (21.4)	1.41 (0.59-1.3.36)			
APRI				0.7		
≤0.5	113	21 (18.6)	1			
0.5 to 1.5	118	19 (16.1)	1.06 (0.51-2.18)			
1.5 to 2.0	15	3 (20.0)	1.16 (0.29-4.66)			
≥2.0	28	3 (10.7)	0.65 (0.17-2.52)			
Missing	69	16 (23.2)	1.55 (0.71-3.36)			
FIB-4 score				0.65		
<1.3	120	19 (15.8)	1			
1.3 to 2.67	102	18 (17.6)	1.37 (0.65-2.88)			
2.67 to 3.25	15	3 (20.0)	1.50 (0.37-6.16)			
>3.25	37	6 (16.2)	1.36 (0.46-4.01)			
Missing	69	16 (23.2)	1.87 (0.85-4.09)			
Alcohol consumption				0.95		
No	112	23 (20.5)	1			
Yes	230	39 (17.0)	0.98 (0.53-1.80)			
Missing	1	-	–			
Reported anxiety/depression signs				<0.001		<0.001
No	301	38 (12.6)	1		1	
Yes	42	24 (57.1)	10.63 (4.72-23.96)		5.95 (2.36-14.98)	
VAS <25^th^ percentile				<0.001		<0.001
No	254	21 (8.3)	1		1	
Yes	83	39 (47.0)	9.45 (4.96-17.97)		7.14 (3.40-15.01)	
Missing	6	2 (33.3)	5.00 (0.82-30.53)		3.41 (0.49-23.66)	

OR: odds ratio; CI: confidence interval; PWID: people who inject drugs; BMI: body mass index; HBV; hepatitis B virus; HCV: hepatitis C virus; ART: antiretroviral therapy; APRI: aspartate aminotransferase to platelet ratio index.

Using mixed effect logistic regression models, the only factors significantly associated with anxiety/depression in univariate and multivariate analysis were reporting pain/discomfort and a VAS score < 25th percentile (Table 4). As compared to those who did not report pain/discomfort signs, those who reported pain/discomfort signs were at higher odds of reporting anxiety/depression (aOR [95% CI]: 7.26 [2.89–18.27]). Those with a VAS score < 25th percentile, as compared to those with a higher score, were at higher odds of reporting anxiety/depression (aOR [95% CI]: 4.05 [1.71–9.62]). Age at enrolment, APRI score at enrolment and alcohol consumption were also considered in the multivariate model, however these factors were not associated with anxiety/depression. When investigating the effect of the Fibrosis-4 (FIB4) score, the category corresponding to a score ranging from 2.65 to 3.25 accounted for 15 participants, none of them reporting anxiety/depression signs. When this category was merged with one or the other adjacent ones, the effect remained not significant (*p* = 0.78 and *p* = 0.34). Regarding hepatitis B virus (HBV) infection, of the 132 participants who were negative, 17 (12.9%) showed anxiety/depression signs. Of the 10 participants who were infected with HBV, none declared signs of depression or anxiety, not allowing us to estimate the effect for this factor.

**Table 4:** Factors associated with anxiety/depression (mixed effect logistic regression).

	N	Anxiety/depression	Crude OR (95% CI)	P	Adj. OR (95% CI)	p
PWID				0.81		
No	94	11 (11.7)	1			
Former	198	20 (10.1)	1.00 (0.42-2.36)			
Drug-substitution prog.	32	5 (15.6)	1.17 (0.33-4.13)			
Current	19	6 (31.6)	1.82 (0.49-6.84)			
Male gender				0.52		
No	19	2 (10.5)	0.59 (0.12-2.97)			
Yes	324	40 (12.3)	1			
Age (years)				0.1		
≤29	14	1 (7.1)	0.58 (0.06-5.60)			
30-39	126	9 (7.1)	1			
40-49	150	23 (15.3)	2.86 (1.09-7.48)			
≥50	53	9 (17.0)	3.27 (0.99-10.82)			
BMI (kg/m²)				0.48		
<18.5	53	8 (15.1)	1.61 (0.63-4.07)			
18.5-25	277	32 (11.5)	1			
≥25	13	2 (15.4)	2.10 (0.33-13.35)			
HBV status			Non estimable			
Negative	132	17 (12.9)				
Positive	10	-				
Not tested	201	25 (12.4)				
HCV viral load				0.36		
Undetected	57	9 (15.8)	1			
Detected	286	33 (11.5)	0.66 (0.27-1.61)			
Ethnicity				0.24		
Thai	204	18 (8.8)	1			
Kinh	94	20 (21.3)	2.75 (0.84-9.08)			
Other	45	4 (8.9)	1.68 (0.39-7.15)			
Marital status				0.83		
Single	67	7 (10.4)	1			
Married/partnered	240	28 (11.7)	1.04 (0.40-2.72)			
Divorced/Separated	30	6 (20.0)	1.69 (0.41-6.93)			
Unknown	6	1 (16.7)	1.78 (0.15-21.49)			
Education level				0.63		
No schooling	23	1 (4.3)	0.44 (0.05-3.96)			
Elementary	92	8 (8.7)	0.90 (0.34-2.35)			
Secondary	148	19 (12.8)	1			
High school or higher	80	14 (17.5)	1.48 (0.63-3.50)			
Time on ART				0.9		
≤60 months	46147140	5 (8.9)	1			
60-120 months		19 (12.9)	1.22 (0.39-3.79)			
>120 months		18 (12.9)	1.31 (0.40-4.28)			
APRI				0.18		
≤0.5	113	16 (14.2)	1			
0.5 to 1.5	118	8 (6.8)	0.61 (0.23-1.62)			
1.5 to 2.0	15	3 (20.0)	1.87 (0.41-8.61)			
≥2.0	28	6 (21.4)	3.17 (0.84-11.97)			
Missing	69	9 (13.0)	1.31 (0.49-3.51)			
FIB-4 score*				0.78		
<1.3	120	15 (12.5)	1			
1.3 to 2.67	102	11 (10.8)	1.24 (0.49-3.15)			
>2.67	52	7 (18.9)	1.66 (0.54-5.10)			
Missing	69	9 (13.0)	1.52 (0.56-4.10)			
Alcohol consumption				0.14		
No	112	12 (10.7)	1			
Yes	230	30 (13.0)	1.80 (0.83-3.94)			
Missing	1	–	–			
Reported pain/discomfort signs				<0.001		<0.001
No	281	18 (6.4)	1		1	
Yes	62	24 (38.7)	11.98 (5.05-28.41)		7.26 (2.89-18.27)	
VAS <25^th^ percentile				<0.001		0.006
No	254	13 (5.1)	1		1	
Yes	83	28 (33.7)	7.58 (3.43-16.76)		4.05 (1.71-9.62)	
Missing	6	1 (16.7)	3.36 (0.27-41.30)		1.65 (0.06-42.37)	

OR odds ratio; CI confidence interval; PWID people who inject drugs; BMI body mass index; HBV hepatitis B virus; HCV hepatitis C virus; ART antiretroviral therapy; APRI aspartate aminotransferase to platelet ratio index.

Factors associated with low self-rated HRQoL were identified using univariate and multivariate logistic regression models (Table 5). Of the 343 patients, 337 (98.2%) self-rated their health and 336 were considered in the multivariate model as one participant did not answer to the question regarding alcohol consumption. Participants who reported pain/discomfort signs and participants who reported anxiety/depression signs presented higher odds of low self-rated HRQoL (aOR [95% CI]: 12.50 [5.63–27.75] and 4.95 [1.99–12.31], respectively). Even after adjusting for these two factors, PWID presented significantly higher odds of low self-rated HRQoL, whether they were former PWID or enrolled in a drug-substitution programme (aOR [95% CI]: 3.07 [1.20–7.87] and 5.88 [1.71–20.21], respectively), as compared to non-PWID, and even higher in current PWID (aOR [95% CI]: 36.88 [8.52–159.67.52.67]). The odds were not significantly different between former PWID and those enrolled in a drug-substitution programme (*p* = 0.19), while they were significantly higher in current PWID when compared to former PWID or those enrolled in a drug-substitution programme (*p* < 0.001 and *p* = 0.013, respectively). Participants with detectable HCV viral load were also at significantly higher odds of low self-rated HRQoL (aOR [95% CI]: 4.00 [1.32–12.12]) as compared to HCV-infected individuals who presented undetectable HCV viral load without having ever received anti-HCV treatment. As compared to the Thai ethnicity, Kinh presented higher odds of low self-rated HRQoL (aOR [96% CI]: 3.08 [1.49–6.37]). Finally, participants who declared alcohol consumption also presented higher odds of low self-rated HRQoL (aOR [95% CI]: 2.49 [1.13–5.47]). Gender, age at enrolment and BMI at enrolment were considered in the multivariate model but were no longer significantly associated with low self-rated HRQoL. In a sensitivity analysis, when PWID status was defined as a binary variable, results were essentially unchanged (supplementary Table 1).

**Table 5:** Factors associated with low self-rated health-related quality of life (VAS score <80; mixed effect logistic regression).

	N	VAS score <80	Crude OR (95% CI)	P	Adj. OR (95% CI)*	P
PWID				<0.001		<0.001
No	94	13 (14.3)	1		1	
Former	198	47 (24.0)	2.10 (1.04-4.26)		3.07 (1.20-7.87)	
Drug-substitution prog.	32	9 (29.0)	2.38 (0.86-6.62)		5.88 (1.71-20.21)	
Current	19	14 (73.7)	13.82 (3.96-48.18)		36.88 (8.52-159.67)	
Male gender				0.043		
No	19	1 (5.3)	0.12 (0.02-0.93)			
Yes	318	82 (25.8)	1			
Age (years)**				0.12		
≤39	139	25 (18.0)	1			
40-49	147	38 (25.8)	1.49 (0.78-2.84)			
≥50	51	20 (39.2)	2.33 (1.04-5.24)			
BMI (kg/m²)				0.17		
<18.5	51	18 (35.3)	1.83 (0.93-3.62)			
18.5-25	273	63 (23.1)	1			
≥25	13	2 (15.4)	0.61 (0.12-3.14)			
HBV status				0.6		
Negative	130	37 (28.5)	1			
Positive	10	2 (20.0)	0.50 (0.09-2.70)			
Not tested	197	44 (22.3)	0.78 (0.41-1.51)			
HCV viral load				0.041		0.014
Undetected	53	7 (13.2)	1		1	
Detected	284	76 (24.8)	2.47 (1.04-5.87)		4.00 (1.32-12.12)	
Ethnicity				<0.001		0.009
Thai	199	31 (15.6)	1		1	
Kinh	93	41 (44.1)	4.11 (1.99-8.49)		3.08 (1.49-6.37)	
Other	45	11 (24.4)	1.78 (0.73-4.33)		2.08 (0.77-5.60)	
Marital status				0.74		
Single	65	14 (21.5)	1			
Married/partnered	236	61 (25.8)	1.24 (0.62-2.50)			
Divorced/Separated	30	7 (23.3)	0.93 (0.30-2.88)			
Unknown	6	1 (16.7)	0.47 (0.05-4.75)			
Education level				0.46		
No schooling	21	4 (19.0)	1.19 (0.35-4.01)			
Elementary	90	19 (21.1)	1.17 (0.59-2.32)			
Secondary	147	32 (21.8)	1			
High school or higher	79	28 (35.4)	1.70 (0.89-3.25)			
Time on ART				0.13		
≤60 months	50	6 (12.0)	0.37 (0.14-0.97)			
60-120 months	124	29 (23.4)	0.81 (0.45-1.46)			
>120 months	163	48 (29.5)	1			
APRI				0.07		
≤0.5	112	35 (31.2)	1	0.32*		
0.5 to 1.5	118	27 (22.9)	0.83 (0.44-1.56)			
1.5 to 2.0	15	7 (46.7)	2.33 (0.73-7.46)			
≥2.0	28	6 (21.4)	0.68 (0.23-2.02)			
Missing	64	8 (12.5)	0.37 (0.15-0.88)			
FIB-4 score				0.26		
<1.3	119	36 (30.2)	1	0.99***		
1.3 to 2.67	102	25 (24.5)	0.92 (0.49-1.75)			
2.67 to 3.25	15	4 (26.7)	0.94 (0.26-3.39)			
>3.25	37	10 (27.0)	1.00 (0.40-2.51)			
Missing	64	8 (12.5)	0.37 (0.16-0.90)			
Alcohol consumption				0.06		0.023
No	110	23 (20.9)	1		1	
Yes	226	59 (26.1)	1.75 (0.97-3.15)		2.49 (1.13-5.47)	
Missing	1	1 (100)	–		–	
Reported pain/discomfort signs				<0.001		<0.001
No	277	44 (15.9)	1		1	
Yes	60	39 (65.0)	9.41 (4.86-18.20)		12.50 (5.63-27.75)	
Reported anxiety/depression signs				<0.001		<0.001
No	296	55 (18.6)	1		1	
Yes	41	28 (68.3)	7.81 (3.60-16.92)		4.95 (1.99-12.31)	

VAS visual analog scale; OR odds ratio; CI confidence interval; PWID people who inject drugs; BMI body mass index; HBV hepatitis B virus; HCV hepatitis C virus; APRI aspartate aminotransferase to platelet ratio index. *Multivariate analysis on 336 participants as the subject with missing data for alcohol consumption was not considered. **category≤29 years and 30 to 39 years were merged as no participant declared a VAS level<80 in the first category.

## Discussion

Overall, in this cohort of HIV-HCV coinfected participants in Vietnam, only a minority of study participants reported issues with their HRQoL prior to HCV therapy. The proportion reporting anxiety/depression signs was 12.2% and was not statistically different between PWID and non PWID. This proportion was in line with that of previous reports in Vietnam^8,9^. Two older studies reported much higher rate of depression^20,25^, however the patients considered in these studies were on ART for 3.5 years in median, which is a lot less than the nearly 10 years in median observed in our study. Importantly, no difference was evidenced in terms of time since ART initiation or ART combination between PWID and non-PWID. Maybe the lower level of depression we observed, as compared to that in previous reports, is explained by a healthy-survivor bias. Yet, the effect of ART duration was investigated but was not associated with anxiety/depression. Our study population consisted only of HIV-HCV co-infected patients. Therefore, we could not investigate whether HCV infection impacted the HRQoL. However, some patients showed signs of active HCV infection through detectable HCV viral load, and hence needed treatment, while some others spontaneously cleared from their HCV infection. The HCV viral load level was however not associated with anxiety/depression. While FIB4 and APRI scores are recognised as indicators of liver damage^21^, these scores were not associated with anxiety/depression. These results suggest that HCV infection might be a silent disease as patients don’t show or feel signs of infection, therefore not weighing on PLHIV mental or physical health. It is also possible that HCV infection is not considered as serious as HIV infection. Additionally, it is possible that HCV is less discussed than HIV in Vietnamese society. It would be interesting to explore what HIV-HCV co-infected patients feel about their HCV infection. The only factors investigated that were associated with reporting anxiety/depression signs were reporting pain/discomfort and a VAS score < 25th percentile.

Overall, 62 (18.1%) of the patients reported pain/discomfort signs. As could be anticipated, this proportion being significantly higher in older patients. The only other factors investigated that were associated with pain/discomfort signs were reporting anxiety/depression signs and a VAS score < 25th percentile.

The EQ-5D-5L scale also tried to capture the general HRQoL through a VAS. Looking at the VAS score, study participants rated their HRQoL with a high score. In median (IQR) the score was 86 (80–91). This result is consistent with another evaluation in PLHIV in Vietnam that showed a very high HRQoL score when on ART^7^, even if the scale used in both evaluations were different making direct comparison complex. Our study participants were on ART for 10 years in median, and the high self-rated HRQoL score may be imputable to healthy survivor bias. Another study in PLHIV in Vietnam evaluated HRQoL using the same scale as in the current evaluation, and participants reported a mean VAS score of 73.6, lower than we observed^8^. Interestingly, the VAS score was lower in those presenting anxiety/depression signs. That study also suggested the positive impact of ART on HRQoL. In another study it was even lower^26^, but this evaluation was conducted in 2012 when conditions for ART initiation and ART combination were different. In that evaluation, the VAS score increased with ART duration.

In our evaluation, although the self-rated HRQoL score was high, the score was significantly lower in PWID than non-PWID, and it was even lower in current PWID as compared to former PWID. Previous studies also reported lower HRQoL in PWID^8,26^. In these latter studies, the median ART duration was much shorter (3 years in mean and around 2 years in median, respectively) while the median ART duration in our study was as long as 10 years, and was not statistically different between PWID and non-PWID. It is striking that, despite the long-time experience with HIV care and ART, PWID still rate their HRQoL lower that non-PWID. The fact that the association between PWID status and lower VAS score persists even after controlling for reporting pain/discomfort, and reporting anxiety/depression suggests that the overall lower HRQoL in PWID is also driven by factors not related to their health status. One can wonder if this represents a worse health level in PWID, or presence of other conditions that we did not investigate. The lower self-rated HRQoL in PWID could also be explained by their lifestyle and directly related to drug use, but this may as well be linked to felt stigma by PWID. Indeed, previous studies in Vietnam concluded that HIV-stigma was associated with deteriorated QoL^27^, but it has been suggested that stigma related to drug-use was more important that HIV-related stigma^10,28^. It is possible that here also, it is stigma felt by PWID that explains the lower HRQoL score seen. This is further supported by the fact that even former PWID reported lower HRQoL score than non-PWID. Unfortunately, we did not investigate felt stigma in our study. Still, initiative to sensitize healthcare teams to help provide secured and welcoming environment for HIV care and services should be implemented. It would be interesting to investigate the HRQoL of PLHIV before and after such an intervention, and to focus on potentially marginalized populations that are PWID, MSM and transgender individuals.

Of note, only five patients reported severe signs in at least one dimension covered by the EQ-5D-5L scale. Interestingly, all these patients were PWID. Although this is a small sample size, this suggest that PWID are more fragile and special attention should be given to their healthcare, especially in the context of HIV infection, as it is crucial to maintain them in care for their own health but also as a public health approach to control the epidemic.

The clinical relevance of the differences observed between PWID and non-PWID warrants consideration. Although minimal clinically important differences (MCIDs) have been proposed for EQ-5D-5L outcomes, MCID thresholds are primarily intended to interpret within-person change over time following an intervention^29–31^, which is not the object here. In the literature, there is no consensus on a threshold that would define low self-rated HRQoL, therefore, we chose an empirical definition using the 25th percentile. The multivariate logistic regression model showed that PWID had significantly higher odds of reported low self-rated HRQoL, as compared to non-PWID, and that odds were even higher in current PWID as compared to former PWID or those engaged in a drug-substitution programme.

As could be expected, reporting anxiety/depression signs was associated with low self-rated HRQoL. This has been reported in another evaluation in Vietnam when using another scale to evaluate HRQoL and depression^9^. Even if depression concerned a small number of participants, caring for mental health should be addressed as it can lead to poorer health outcomes^32,33^. Those who reported pain/discomfort signs also self-rated the HRQoL at a lower level. In this population, reporting pain/discomfort signs, reporting anxiety/depression signs and a low VAS score were strongly associated. It therefore seems plausible that the lower self-reported HRQoL observed is partly driven by pain/discomfort and anxiety/depression symptoms. Although most participants reported only slight to moderate problems in these domains, these symptoms were nevertheless associated with higher odds of low self-rated HRQoL. Interventions targeting pain management and mental health could thus help improve HRQoL.

As with depression or pain, the self-rated HRQoL level estimated through the VAS was not influenced by the level of liver damage expressed by the FIB4 or APRI scores. This time however, those presenting detectable HCV viral load were at higher odds of low self-rated HRQoL score. Maybe chronic HCV infection impacts the general HRQoL. However, this association was not evidence with pain/discomfort nor with anxiety/depression. A report in Canada, in HIV-HCV co-infected individuals reported an increase in the HRQoL score after successful HCV treatment^34^, which also suggests that HCV infection impacts the HRQoL.

Vietnam is a multiethnic country comprising 54 different ethnic groups. In our study, the most common ethnic group was Thai followed by Kinh, this latter being the main ethnic group in Vietnam. Ethnic minorities are more often found in remote settings, and appear to be less rich than Kinh ethnic group^35^. Although this report dates back from 2013, in general, ethnic minorities are believed to have lower use of health services in Vietnam^35^. More recently, it was shown that in pregnant women, access to care differed between ethnic groups^36^. Kinh ethnicity was associated with higher odds of reporting low self-rated HRQoL, as compared to the Thai ethnicity. Maybe Kinh individuals, who belong to the ethnic majority, perceive a larger gap between what they expect and what they experience, leading to low self-rated HRQoL. On the contrary, other ethnic minorities may have more modest expectations leading to higher self-rated HRQoL.

The quantitative aspect of our study, in two remote provinces, indicated that PWID status and Kinh ethnicity were associated with low self-rated HRQoL. We could not investigate further the reasons why these groups experienced lower HRQoL. As part of the current study, we plan to implement one-to-one in-depth interviews after HCV treatment. Stigma, related to HIV and to drug use, will both be questioned during this phase in an attempt to better understand our results. Ethnic context will also be discussed during that phase, as well as other topics such as HIV care and HCV infection to gain a deeper understanding and interpretation of these results.

This study has some limitations, however. The EQ-5D-5L scale was administered to participants by a member of the clinical team, rather than being completed directly by the participants themselves. This may have led to underreporting or underestimation of the problems encountered by the PLHIV surveyed, through desirability bias. This may also have increased the VAS score, although very high VAS scores have been previously reported^7^. Still, PWID declared a significantly lower VAS score than non-PWID.

One can wonder whether the various questions about the HRQoL and the VAS have been correctly understood in this very decentralised setting. However, the level of education was systematically investigated, and it was never associated with the outcomes. Moreover, the EQ-5D-5 L scale is validated in Vietnamese language and has been used in a large panel of populations.

Regarding the VAS, we empirically defined a low score based on the 25th percentile. No threshold is recognised to allow easier comparison between studies. Tentatives have been conducted^37^, but could it be independent from the context?

The EQ-5D-5L is an easy-to-use tool to describe the HRQoL. However, unlike other instruments, it is not recognised as a discriminatory screening tool used in clinical practice to identify those who may experiment mental health issues and could benefit from proper mental health care. An evaluation in Vietnam showed reduced perceived stigma and improved health when mental health issues were care for in PWID^38^. In the context of HIV care, simple, validated mental health screening scales should be introduced and used more systematically to detect those in need of further psychological assessment and support. A pilot randomized trial implemented in Vietnam evaluated an adapted friendship bench intervention, a low-cost mental health program in which trained lay counselors provide brief, structured problem-solving therapy to treat depression and anxiety, delivered either by health professionals or by peer counsellors^39^. Results were very promising, showing reduction in mental disorders and suggesting improved retention in HIV care. High attendance at counselling sessions over a 6-month period also indicates that patients perceived the intervention as beneficial. However, this approach requires systematic identification of those with mental disorders, and sufficient work force capacity to provide counselling and support. Nevertheless, medical doctors and counsellors involved in the trial expressed confidence that this low-cost approach could be scaled up and implemented in other HIV care sites^40^. In Vietnam, PWID status is an information collected in routine HIV care, and we therefore believe that PWID were correctly identified in our study. When questioned about drug use at study enrolment, most participants reported having quitted drug use. One can’t exclude that some participants declared having quitted drug-use to preserve a positive self-image or to conform to what they perceive as socially expected. The case-report form did not capture date of last drug use, so we were unable to cross information in order to better define sub-PWID status. A gradient in self-rated HRQoL across PWID categories was observed, lowest among current PWID, intermediate among those enrolled in a drug substitution programme and former PWID, and highest in non-PWID. Although not anticipated, this dose–response pattern supports the accuracy of PWID classification.

## Conclusions

Our results suggest that PWID face more physical and psychological issues than other participants. The prevalence extent of the problems met are difficult to ascertain from our study, but screening patients to identify those who need more specific support or attention would be beneficial. Offering integrated mental health screening and care could also benefit these patients, but then the question of integrating mental health care in the national health insurance must be questioned and addressed.

## Supplementary Information

Below is the link to the electronic supplementary material.

Supplementary Material 1

## Data Availability

The datasets used and/or analysed during the current study are available from the corresponding author on reasonable request.

## Abbreviations

APRI: Aspartate aminotransferase to platelet ratio index
ART: Antiretroviral therapy
BMI: Body mass index
FIB-4: Fibrosis-4
HBV: Hepatitis B virus
HCV: Hepatitis C virus
HIV: Human immunodeficiency virus
IQR: Inter quartile range
MSM: Men who have sex with men
OR: Odds ratio
PLHIV: People living with HIV
PWID: People who inject drugs
QoL: Quality of life
VAS: Visual analog scale
