# Supplementary material for: Injecting drug use worsens the quality of life in HIV-HCV co-infected patients in Vietnam
Source: Sci Rep. 2026 Apr 9;16:16192. doi: 10.1038/s41598-026-46919-7 (PMC13201638; doi:10.1038/s41598-026-46919-7)
Supplement: Supplementary file 1 — Supplementary Material 1 [file 41598_2026_46919_MOESM1_ESM.docx]

**Supplementary Table 1**: Factors associated with low self-rated health quality (VAS-score <80; mixed effect logistic regression)

|  | N | VAS-score <80 | Crude OR (95% CI) | P | Adj. OR (95% CI)* | P |
| --- | --- | --- | --- | --- | --- | --- |
| PWID  No  Yes | 91  246 | 13 (14.3)  70 (28.5) | 1  2.61 (1.31-5.19) | 0.006 | 1  4.29 (1.71-10.74) | 0.002 |
| Male gender  No  Yes | 19  318 | 1 (5.3)  82 (25.8) | 0.12 (0.02-0.93)  1 | 0.043 |  |  |
| Age (years)**  ≤39  40-49  ≥50 | 139  147  51 | 25 (18.0)  38 (25.8)  20 (39.2) | 1  1.49 (0.78-2.84)  2.33 (1.04-5.24) | 0.12 |  |  |
| BMI (kg/m²)  <18.5  18.5-25  ≥25 | 51  273  13 | 18 (35.3)  63 (23.1)  2 (15.4) | 1.83 (0.93-3.62)  1  0.61 (0.12-3.14) | 0.17 |  |  |
| HBV status  Negative  Positive  Not tested | 130  10  197 | 37 (28.5)  2 (20.0)  44 (22.3) | 1  0.50 (0.09-2.70)  0.78 (0.41-1.51) | 0.60 |  |  |
| HCV viral load  Undetected  Detected | 53  284 | 7 (13.2)  76 (24.8) | 1  2.47 (1.04-5.87) | 0.041 | 1  4.52 (1.51-13.51) | 0.007 |
| Ethnicity  Thai  Kinh  Other | 199  93  45 | 31 (15.6)  41 (44.1)  11 (24.4) | 1  4.11 (1.99-8.49)  1.78 (0.73-4.33) | <0.001 | 1  3.20 (1.58-6.48)  2.40 (0.94-6.15) | 0.004 |
| Marital status  Single  Married/partnered  Divorced/Separated  Unknown | 65  236  30  6 | 14 (21.5)  61 (25.8)  7 (23.3)  1 (16.7) | 1  1.24 (0.62-2.50)  0.93 (0.30-2.88)  0.47 (0.05-4.75) | 0.74 |  |  |
| Education level  No schooling  Elementary  Secondary  High school or higher | 21  90  147  79 | 4 (19.0)  19 (21.1)  32 (21.8)  28 (35.4) | 1.19 (0.35-4.01)  1.17 (0.59-2.32)  1  1.70 (0.89-3.25) | 0.46 |  |  |
| Time since HIV diagnosis  ≤60 months  60-120 months  >120 months | 50  124  163 | 6 (12.0)  29 (23.4)  48 (29.5) | 0.37 (0.14-0.97)  0.81 (0.45-1.46)  1 | 0.13 |  |  |
| APRI  ≤0.5  0.5 to 1.5  1.5 to 2.0  ≥2.0  Missing | 112  118  15  28  64 | 35 (31.2)  27 (22.9)  7 (46.7)  6 (21.4)  8 (12.5) | 1  0.83 (0.44-1.56)  2.33 (0.73-7.46)  0.68 (0.23-2.02)  0.37 (0.15-0.88) | 0.07  0.32* |  |  |
| Fibrosis-4 score  <1.3  1.3 to 2.67  2.67 to 3.25  >3.25  Missing | 119  102  15  37  64 | 36 (30.2)  25 (24.5)  4 (26.7)  10 (27.0)  8 (12.5) | 1  0.92 (0.49-1.75)  0.94 (0.26-3.39)  1.00 (0.40-2.51)  0.37 (0.16-0.90) | 0.26  0.99*** |  |  |
| Alcohol consumption  No  Yes  Missing | 110  226  1 | 23 (20.9)  59 (26.1)  1 (100) | 1  1.75 (0.97-3.15)  - | 0.06 | 1  1.83 (0.88-3.83)  - | 0.10 |
| Reported pain/discomfort  No  Yes | 277  60 | 44 (15.9)  39 (65.0) | 1  9.41 (4.86-18.20) | <0.001 | 1  19.80 (4.52-22.27) | <0.001 |
| Reported anxiety/depression  No  Yes | 296  41 | 55 (18.6)  28 (68.3) | 1  7.81 (3.60-16.92) | <0.001 | 1  5.78 (2.26-14.83) | <0.001 |

VAS: visual analog scale; OR: odds ratio; CI: confidence interval; PWID: people who inject drugs; BMI: body mass index; HBV; hepatitis B virus; HCV: hepatitis C virus; APRI: aspartate aminotransferase to platelet ratio index
